# H5P

**DOI:** 10.5195/jmla.2021.1204

**Published:** 2021-04-01

**Authors:** Juliana Magro

**Affiliations:** 1 juliana.mangro@nyulangone.org, Education and Research Librarian, NYU Langone Health, NYU Grossman School of Medicine, New York, NY

## INTRODUCTION

H5P is an open-source authoring tool used to design interactive educational tasks [1]. It allows educators to create content such as presentations, quizzes, games, and even interactive e-books. H5P's ease of use is reflected by its widespread adoption: since it was first launched seven years ago, it has been used by more than 200,000 websites [2]. In addition, almost 18 million users registered an account on their community website [3], and more than 700 organizations adopted H5P's Software as a Service (SaaS) [2]. Its success shows that this versatile and uncomplicated tool can be used across communities all over the world.

Using a self-hosting plugin extension, creators can build new content directly on their web browsers (Chrome or Internet Explorer, for example) at no cost. H5P also offers a subscription service for hosting, support, and detailed student reporting. An added benefit of this tool is that all content types allow for attribution of metadata, benefiting creators and users in two ways. First, it is possible to properly credit other creators when reusing or adapting content. Second, authors choose how to license their creations; they can opt for traditional copyright, Creative Commons licenses, or public domain dedication. This removes any ambiguity when it comes to sharing and adaptations and makes H5P an excellent collaborative tool.

## CONTENT

**Figure 1:**
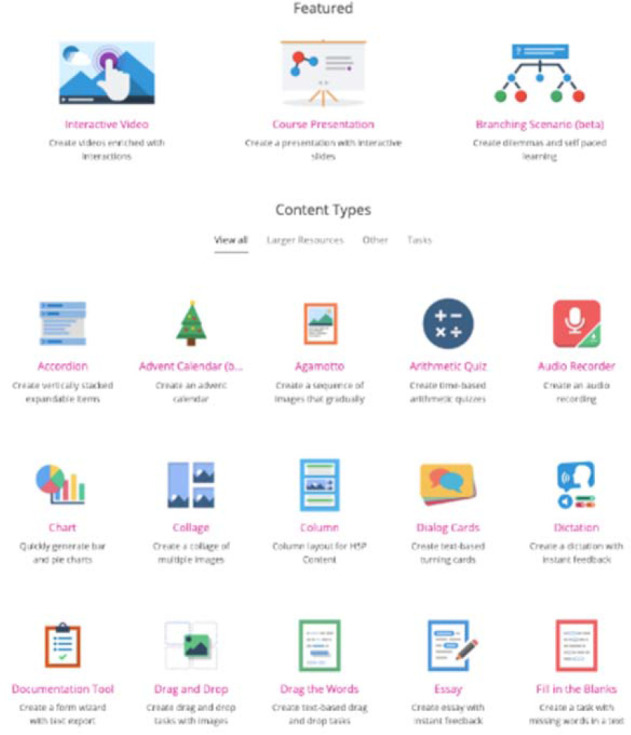
Snapshot of content types, available at https://h5p.org/.

Currently, users can create more than forty types of interactive learning instruments. The developers continue to create new content, and other tasks are expected to be released soon. Content ranges from interactive videos, course presentations, e-book creators, and image hotspots to branching scenarios, flashcards, and a variety of quizzes (single- and multiple-choice, summary, quiz set, fill-in-the-blanks, essay, etc.). Figure 1 shows a sample of content types.

## INTENDED AUDIENCE

To create any of H5P's content types, simply access a website where H5P is installed (see the section below for more details) [4]. This ease of use, coupled with the variety of content, makes H5P easily accessible to educators at any level. For instance, medical educators might use one or more of the following: image hotspots for anatomy annotation; interactive videos enriched with many types of questions, images, tables, and additional text; course presentations for interactive slides; and even branching scenarios for games or dilemmas.

In addition to using activities in classes, librarians can embed tasks into a learning management system (LMS) or into LibGuides. Moreover, H5P is an effective tool for those creating e-books in WordPress-powered platforms. With the free H5P plugin for WordPress, many authors now include built-in quizzes in their e-books, allowing readers to test their knowledge and practice questions with instant feedback.

## INTEGRATIONS

There are three ways to use H5P: through a direct link, via LTI (learning tools interoperability), and using plugins. The first two services are provided via H5P.com and require a subscription; the third is free to use with plugins in Drupal [5], WordPress [6], and Moodle [7]. Since this is an open-source tool, there are also plugins for other platforms made by community members; however, the aforementioned are the only ones developed and maintained by the H5P Core Team.

Although H5P lists integrations for four learning management systems, they report that some users have used H5P.com via LTI in other systems without any trouble [8].

Table 1 compares the different types of integrations available for H5P.

**Table 1:** Integrations and features for H5P [8].

Mode of integration and platform/Features		Hosting	Reporting	Support	Maintained by the core team	Up to Date	Subscription required
*Direct link or embed*	*H5p.com*	Yes	No	Yes	Yes	Yes	Yes
*LTI integration*	*Canvas*	Yes	Yes	Yes	Yes	Yes	Yes
	*Brightspace*	Yes	Yes	Yes	Yes	Yes	Yes
	*Blackboard*	Yes	Yes	Yes	Yes	Yes	Yes
	*Moodle*	Yes	Yes	Yes	Yes	Yes	Yes
*Free plugin*	*Drupal*	No	No	No	Yes	Yes	No (free)
	*Moodle*	No	Some reporting	No	Yes	Yes	No (free)
	*WordPress*	No	No	No	Yes	Yes	No (free)

As shown in Table 1, the advantages of using H5P.com are hosting, reporting, and support from the H5P team. The team also intends to make a few tools available exclusively to paid subscribers, such as live quiz tools. As of July 2020, more than 700 organizations were using H5P.com [2].

## PRICING LEVELS

### Free plugins

As previously mentioned, the plugins for Drupal, WordPress, and Moodle are free.

### H5P Software as a Service (SaaS)

H5P.com SaaS is offered as a tiered module based on a limited number of authors or as an enterprise for all faculty members at an institution. Author-based licenses can be paid monthly or annually and start at $57/month or $570/year [9]. Enterprise licenses allow all faculty members to author content and are based on the number of students within the organization. Quotes are presented upon request.

## USABILITY AND ACCESSIBILITY

As mentioned, there is no need to install any local software to use H5P—authors create and publish content directly on the web browser. This makes H5P simple to use, since it does not require advanced knowledge of coding or any other technical skill. As a result, users with a wide range of digital literacy skills can create a variety of content types. Moreover, authors can learn how to use any of their content types through detailed tutorials [11].

**Figure 2:**
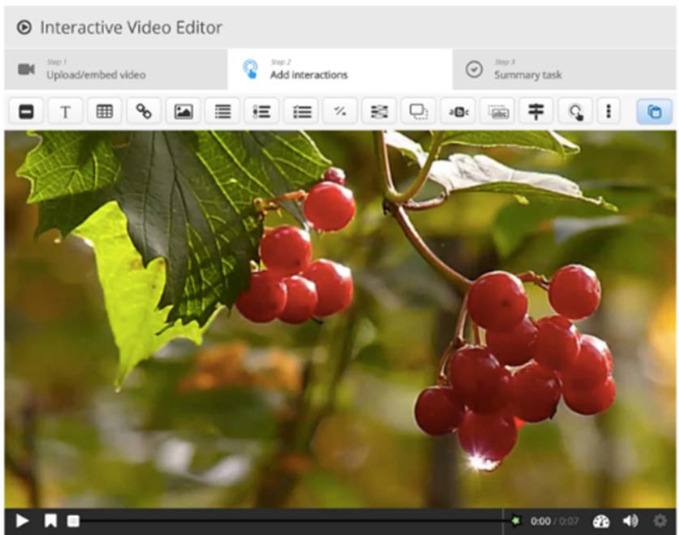
Example of interactive video tutorial [12].

To illustrate this, Figure 2 shows the three steps required to create an interactive video: (1) upload or embed video, (2) add interaction, and (3) summary task. All interactions (multiple-choice questions, free text entry, drag and drop, fill in the blank, drag text, crossroads, etc.) are available as buttons on the top bar. This content also allows for extensive customization with the addition of bookmarks and behavioral settings.

In terms of accessibility, H5P.com maintains a list of all content items and indicates if they are accessible [13]. They state that all content has been tested against Web Content Accessibility Guidelines (WCAG) 2.1 [14]. Based on their tests, most content types are accessible, are supported by all web browsers, and are maintained by the H5P Core Team. In cases where a content type is not accessible, they indicate that the core team is either actively fixing the issues observed or do not recommend using this content type.

Overall, the documentation provided is clear and transparent. It is worth noting that H5P maintains content types that will more easily be used in educational settings. A few examples of content no longer supported are Twitter User Feed, Speak the Words, Audio Recorder, and Personality Quiz. For a full list, check H5P's documentation [13].

## INTELLECTUAL PROPERTY, METADATA, AND SHARING

When creating educational content through software, a frequent concern is ownership of the creation. With H5P, content is owned by its creators. If you subscribe to H5P.com and terminate your subscription, you can still download and keep your content as an H5P package. You can then host it again freely via plugin or after you reactivate your subscription.

H5P also facilitates the processes of adding metadata. In addition to the title, authors can choose how to license their creation and add metadata about the source, authors, authors’ roles, a changelog, and any other information desired. Furthermore, authors are encouraged to properly attribute any other resource added to their tasks (like pictures or videos).

In terms of sharing, authors have the option to allow users to download or embed the content created. For those using the paid subscription alternative, there is an additional option to set the status as unpublished, protected, or public. These features facilitate sharing and ensure that creations are used in accordance with the author's intent. Although the tool itself is conducive to collaboration, there is not a centralized repository where people can search for tasks to be reused and adapted. That is why the H5P Core team is now tasked with the creation of the H5P Open Educational Resources (OER) Hub [15]. The hub will allow people to share open content and, in turn, search and repurpose content already created.

## SUMMARY

H5P is a powerful tool for creating and sharing rich interactive learning instruments. In addition to the clear applicability to various educational settings, there are many other advantages to using H5P, including the following:

- authors retain ownership over their creations;
- Joubel, the company behind H5P.com, is creating an Open Educational Resources Hub where authors can collaborate and share open content;
- H5P is an open software used worldwide;
- H5P.com provides support and student reports for those who subscribe to the service; and
- the documentation available on their websites is detailed and transparent.

In sum, H5P is a robust and easy-to-use tool for educators to enrich their classes and to create instant feedback activities in modules, open textbooks, and LibGuides.
